# Psychometric Properties of the Greek Version of the Autism Parenting Stress Index (APSI) among Parents of Children with Autism Spectrum Disorder

**DOI:** 10.3390/diagnostics13203259

**Published:** 2023-10-19

**Authors:** Angelos Papadopoulos, Stefania Fouska, Dionysios Tafiadis, Nikolaos Trimmis, Panagiotis Plotas, Vassiliki Siafaka

**Affiliations:** 1 Department of Speech and Language Therapy, School of Health Rehabilitation Sciences, University of Patras, 26504 Patras, Greece; nicktrimmis@upatras.gr; 2 General Children’s Hospital of Patras “Karamandaneio”, 26331 Patras, Greece; 3 Department of Education, School of Education, University of Nicosia, Nicosia 2417, Cyprus; stefaniafouska@yahoo.gr; 4 Department of Speech and Language Therapy, School of Health Sciences, University of Ioannina, 45110 Ioannina, Greece; tafiadis@uoi.gr (D.T.); siafaka@uoi.gr (V.S.); 5 Laboratory of Primary Health Care, School of Health Rehabilitation Sciences, University of Patras, 26504 Patras, Greece

**Keywords:** autism, APSI, parenting stress, validation, children, Greek language, ASD

## Abstract

(1) Background: This study aimed to validate the Greek version of the Autism Parenting Stress Index (APSI) among parents of children with ASD. (2) Methods: The translated version was administered to 113 parents (Male: 12, Female: 101, 39.24 years old, SD 6.70, age range, 25–58) of children diagnosed with ASD and 127 parents (Male: 24, Female: 103, 41.08 years old, SD 6.22, age range: 27–56) of typically developing children. (3) Results: Significant differences between the APSI total scores and three domains between groups were observed. Although the initial factor structure could not be replicated, the APSI’s internal consistency was excellent (a = 0.914), with a high positive item–total correlation (0.900–0.917). The APSI’s test–retest reliability was excellent, showing an ICC equal to 0.922 [95%, CI: 0.900–0.940]. The APSI’s total score cut-off point was equal to 12.00 (AUC 0.845, *p* < 0.001) with a sensitivity of 0.839 and 1-specificity of 0.220. A principal component analysis of the 13 items, using varimax rotations, identified three factors, which explained approximately 45.8% of the overall variance. (4) Conclusions: The Greek version of the APSI exhibited discriminant validity for measuring parents of children with ASD. Greek health professionals can use it to assess the stress experienced by parents of children with ASD.

## 1. Introduction

As a neurological developmental disorder, autism spectrum disorder (ASD) is frequently identified in children between the ages of two and three years [1]. ASD has a varying prognosis because of its nature, but it is widely accepted as a lifelong condition. Speech and language, sociability, sensory perception, cognitive awareness, health, and physical behavior deficiencies are characteristics of an individual with ASD [1,2]. According to the literature, all the characteristics mentioned above of ASD are significant sources of parenting stress and mental health problems in parents of children with ASD [1,3].

In general, stress describes the individual’s normal physical and mental response to a threat or a demanding situation that they consider to be beyond their ability to cope [4]. Researchers have defined parenting stress as a negative psychological reaction to parenting responsibilities [3,5]. Everyday tasks and responsibilities, such as feeding, bathing, and transporting their child, can cause parents to feel exhausted and frustrated even more in cases where the children are even-tempered [5]. The strain of parenting can have an adverse effect not only on an individual’s well-being but also on the capacity of their family to provide helpful interactions for one another [6]. Studies [5,7] have indicated a significant association between parenting stress and child behavior problems. Research indicates that overall, parental stress relates to both the child’s outward and the parent’s inward manifestations of behavior problems [5,7]. All families may be affected by the stress of parenting; however, certain factors as mediator variables influence the relationship between parenting stress and child behavior problems, such as demographic factors (e.g., child gender, age, and race) [5].

### 1.1. Parenting Stress in ASD

The daily life of a family includes many stressful experiences that affect parents and their children, a fact that becomes even more evident in exceptional cases, such as when the child suffers from a disorder. The family may experience a crisis or be dysfunctional if a child has a chronic illness or a disability [4]. According to the literature, compared to parents of typically developing children or other clinical groups, parents at risk for behavior problems, such as those with intellectual disabilities, autism, developmental delay, or genetic syndromes, experience higher levels of parenting stress [1,4,5,8,9,10,11,12,13,14,15,16]. Risk factors that can contribute to increased parenting stress in parents of children with ASD include the level of cognitive development of the child; the temperament of the child, as well as learned patterns of defiance, non-compliance, and demanding behaviors [10,11]; the severity of the core features of autism [12]; and the parents’ perceptions of own parenting [6]. Recent data revealed significantly higher levels of mothers self-reported parental stress and coping, as often they are the primary caregivers, compared with fathers [12,17]. Regarding the relative evidence from Greece, recent studies [3,18,19,20] concluded that the parents of children diagnosed with an autistic spectrum disorder experience negative emotions, which derive from the severity of their child’s health condition, the level of functioning, but also from other social and economic factors [18,19].

Parenting stress impacts parents and children and may also influence the parent–child relationship. It has been linked with increased emotional distress in parents, a lower quality of parenting behavior, and child behavioral and developmental maladjustment [21]. Furthermore, in parents of children with ASD, parenting stress is associated with worsened symptoms, higher sensitivity toward negative parenting behavior, and the exacerbation of children’s socio-emotional dysfunction [22].

### 1.2. Measurements of Parenting Stress in ASD

The most widely used instruments in ASD research for measuring parenting stress are the Parenting Stress Index-Short Form (PSI-SF) [23] and the Questionnaire on Resources and Stress (QRS) [24]. In the Greek population, a recent study [25] in mothers of children with ASD has shown high internal consistency for PSI-SF (Cronbach α was 0.82, 0.86, and 0.88 for the Parent-Child Dysfunctional Interaction (P-CDI), Parental Distress (PD), and Difficult Child (DC), respectively). In addition, the QRS was validated in Greek mothers and fathers of children with ASD, intellectual disability, and learning disorders as well as those of typically developing children, showing good internal consistency, split-half reliability, and coefficient of stability [26].

However, a recent systematic review indicated that scales estimating parenting stress are often improperly implemented and do not accord with the group studied [27]. Among these scales, the Parenting Stress Index (PSI), designed for parents of children between 3 months and ten years old, has been utilized among parents of children with disabilities and those older than the recommended age range [27]. This could cause problems as the scale may not capture the unique factors impacting stress levels in parents raising a child with a disability or ASD, while also not capturing the stressors associated with the child’s respective age. Thus, it is necessary to review the currently utilized scales to ensure they are suitable for parents of children with ASD [27].

Regarding the Greek population, all evidence about parenting stress experienced by parents of children with ASD comes from general questionnaires, which do not include questions concerning the specific characteristics of children with autism. In addition, to the best of our knowledge, no specific autism-related instrument is available in the Greek language. One of the most widely used specific scales for the assessment of parental stress among parents of children with ASD is the Autism Parenting Stress Index (APSI) [14], which is designed to measure the parenting stress as perceived by parents of young children with ASD and the factors causing this stress.

Considering the above, the study aimed to translate and validate the Greek version of APSI, a simple and valid standardized scale to measure parenting stress, considering the complex core and co-morbid symptoms seen in young children with ASD. Effective screening for parenting stress could form the basis for the organization of support services for those suffering from higher stress levels.

## 2. Materials and Methods

### 2.1. Participants

The parents of children with ASD were recruited from a public center of differential diagnosis and support for children with special educational needs, a general pediatric hospital, and public and private speech and occupational therapy centers in Greece. The average age of the parents of children with ASD was 39.24 (SD 6.70) [age range, 25–58] and 41.08 (SD 6.22) [age range, 27–56] for the parents of the TD group. In addition, the average age of the children with ASD was 7.70 (SD 4.40) [age range 2–27] and 7.77 (SD 5.15) [age range 2–22] for the children in the TD group. The inclusion criteria were as follows: (a) diagnosis of ASD in the child, (b) non-participation of parents in a previous psychoeducational intervention program, (c) absence of other family members with a disability, (d) ability of the caregiver to understand and complete the questionnaires, and (e) provision of direct care to the child. All the children with ASD had already received their diagnosis from a child psychiatrist before participating in the study. The diagnosis was announced in person, and the doctor gave the parents all possible information, support, and guidance. The samples’ criteria level was according to DSM-5 [28].

Of the 123 cases that met the inclusion criteria, 2 declined to participate in the study, and 8 cases were excluded from the analysis because of missing data. The control group met the exact same eligibility requirements as the ASD group (except for the ASD diagnosis). In addition, the control group should have a similar age range to the autism group, for both parents and children. Furthermore, the recruitment occurred after an open call to the community via social media. All participants were informed about the purpose of this study, full written consent was obtained from the participants before the study, and the protection of participants’ privacy and the confidentiality of the data were ensured.

### 2.2. Instrument Description of Autism Parenting Stress Index (APSI)

The APSI is specific in identifying parenting stress associated with core symptoms and comorbidities of children diagnosed with ASD. It contains 13 common experiences that parents of children with ASD frequently encounter. The internal consistency (Cronbach’s alpha) of the initial version of the APSI was calculated α = 0.82 for children with ASD. The index asks the parent to choose how stressful each item is for them using a five-point Likert scale ranging from 0 (not stressful) to 4 (so stressful sometimes we feel we cannot cope). The total score ranges from 0 to 52; higher scores indicate greater parenting stress. The APSI [14] includes three domains: (a) core autism symptoms (questions: 1,2, 11, 12, 13), (b) co-morbid behaviors (questions: 3, 4, 5, 6), and (c) co-morbid physical issues (questions: 7, 8, 9, 10). The APSI was translated and validated for English, Spanish, and Chinese populations [14,29,30]. The APSI translation in Greek was carried out according to the guidelines set by the minimal translation criteria from the Scientific Advisory Committee (SAC) of the Medical Outcomes Trust [31]. The minimal translation criteria are outlined as follows. The APSI was assigned to two native speakers with knowledge in the field who were proficient in English. A Greek version was created, and a professional bilingual translator back-translated the version into English. The back-translation was reviewed, and cognitive debriefing procedures were performed. The final version of the APSI was submitted to a pilot study. Finally, a revision was made under the guidance of the expert.

### 2.3. Statistical Analysis

All variables were checked for normality using the Kolmogorov–Smirnov and Shapiro–Wilk tests. The descriptive variables reported included means (*M*) and standard deviations (*SD*). The *t*-test was used for the independent groups, and the one-way ANOVA was used for the multiple groups’ comparisons. The estimation of cut-off values for the APSI was computed through a receiver operating characteristics (ROC) curve analysis. The optimal cut-off values were determined using the robust and effective Youden Index. The Cronbach’s alpha coefficient and the split-half reliability coefficient technique were used to evaluate the internal consistency of the Greek version of the APSI. The interclass correlation coefficient (ICC) was used to flag significant correlations and for test–retest reliability.

A pairwise Spearman rho correlation coefficient was also calculated between the 3 factors from the PCA and the CFA analysis. A set of observed variables was computed using the principal component analysis (PCA) and confirmatory factor analysis (CFA) to be represented in terms of fewer variables (domains). In particular, the PCA is frequently used as a dimensionality reduction technique to shrink the size of a huge data set while retaining the majority of its information. Following the detailed analysis, we first verified that the 13 separate items’ correlations had an absolute value of less than 0.8; items with a correlation absolute value of more than 0.8 had to be excluded from the analysis. To evaluate the factorability of our data, the Kaiser–Meyer–Olkin (KMO) and Bartlett’s test of sphericity were applied. The number of main components employed was then investigated using a scree plot, and the PCA was then carried out.

In this stage, we pre-determined the factor structure and confirmed the structure that the EFA first discovered, whereas the CFA inherited many of the same notions from the EFA. For the CFA, we utilized the robust maximum likelihood estimator for model fitting. We employed the CFI, TLI, and RMSEA as the fit measures for our CFA model. CFI stands for Comparative Fit Index, and values range from 0 to 1 (numbers above 0.70 suggest a good fit). The Tucker Lewis Index (TLI), which ranges from 0 to 1, also indicates a strong fit for values higher than 0.70. The root mean square error of approximation (RMSEA) denotes a model that closely fits the data and was set at a *p*-value > 0.05.

The significance was set at *p* < 0.05 and was two-tailed. Statistical analyses were performed using the IBM SPSS statistical software (version 19.0, Armonk, NY, USA) and the Jamovi software [The Jamovi project (2023). Jamovi. (Version 2.4) [Computer Software]] [32].

## 3. Results

The two groups were similar in age, gender, and years of education. All samples’ demographic data are summarized in Table 1. Starting with the between-group comparisons as regards the APSI questionnaire total score, the analysis showed that the parents’ group of ASD children had statistically significantly higher scores than the parents’ group of TD children [t (238) = −9.849, *p* < 0.001]. Likewise, statistically significant differences were observed for the three factors of the APSI questionnaire, notably: (a) for the “Core autism symptoms” [t (238) = −13.823, *p* < 0.001], (b) for the “Co-morbid behaviors” [t (238) = −5.281, *p* < 0.001], and (c) for the “Co-morbid physical issues” [t (238) = −4.409, *p* < 0.001]. The between-group comparisons and relevant results are presented in Table 2.

The one-way ANOVA method was used to explore the main group effect between the parents’ group of TD children, the parents’ group of level 1 (requiring support) ASD children, the parents’ group of level 2 (requiring substantial support) ASD children, and the parents’ group of level 3 (requiring very substantial support) ASD children. The analysis returned for the APSI total scores [F (3, 235) = 32.552, *p* < 0.001], the “Core autism symptoms” total score [F (3, 235) = 65.693, *p* < 0.001], the “Co-morbid behaviors” total score [F (3, 235) = 9.733, *p* < 0.001], and the “Co-morbid physical issues” total score [F (3, 235) = 8.854, *p* < 0.001]. In all measurements, the parents’ group of TD level 2 children had the higher scores (Table 3). The one-way ANOVA method was used to explore the main group effect between parents of children with ASD. No statistically significant differences were observed for the APSI total scores [F (2, 109) = 0.644, *p* = 0.527], the “Core autism symptoms” total score [F (2, 109) = 2.155, *p* = 0.121], the “Co-morbid behaviors” total score [F (2, 109) = 0.598, *p* = 0.552], and the “Co-morbid physical issues” total score [F (2, 109) = 0.519, *p* = 0.596].

### 3.1. Receiver Operating Characteristics Analysis for the APSI

**Figure 1:**
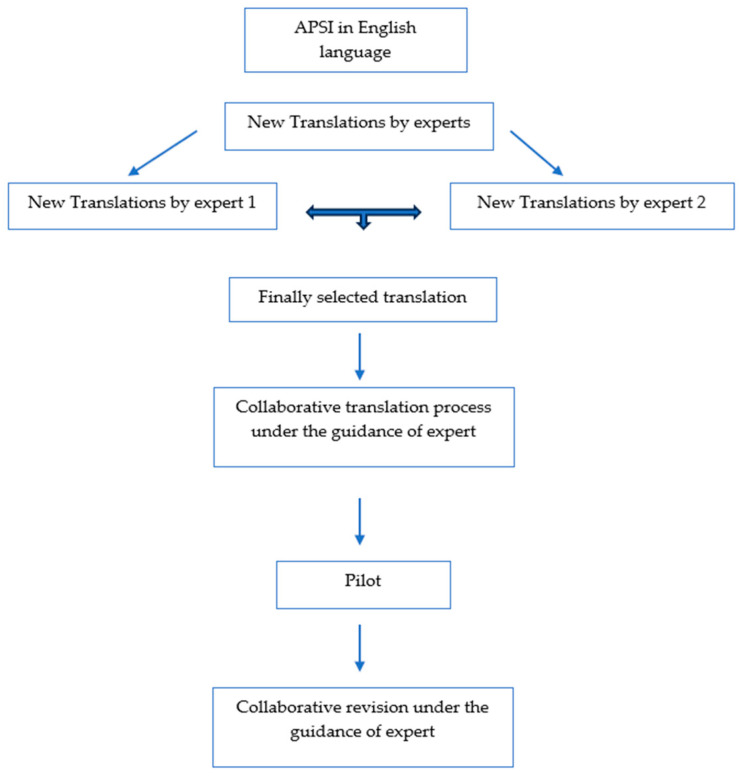
Translation process.

**Figure 2:**
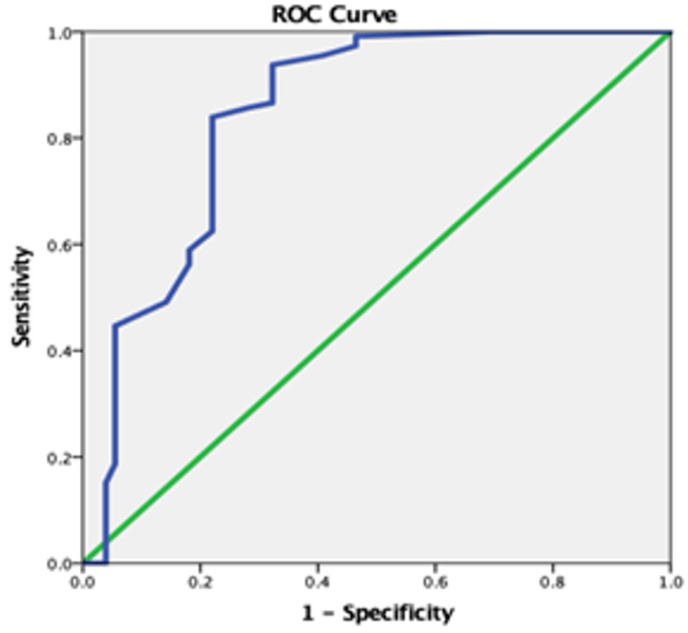
Receiver operating characteristics (ROC) curve for the APSI total score—between parents’ group of ASD children and parents’ group of TD children.

**Figure 3:**
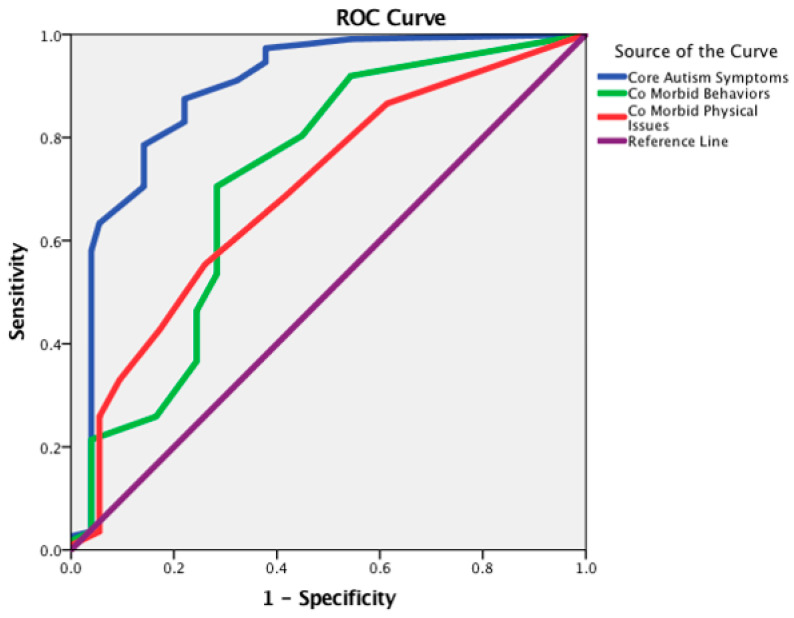
Receiver operating characteristics (ROC) curve for the three factors of APSI—between parents’ group of ASD children and parents’ group of TD children.

The cut-off points of the APSI total score and its three factors were computed using an ROC analysis (Figure 1). A statistically significant positive discrimination between the parents’ group of ASD children and the parents’ group of TD children was revealed [AUC 0.845 (95% CI: 0.794–0.895), *p* < 0.001]. According to the Youden Index, the optimal cut-off point was equal to 12.00 with a sensitivity of 0.839 and 1-specificity of 0.220 for the APSI questionnaire total score (Figure 2). Additionally, a ROC analysis revealed statistically significant positive discrimination for the APSI’s three factors: (a) “Core autism symptoms” [AUC 0.892 (95% CI: 0.850–0.934), *p* < 0.001] optimal cut-off point 6.00 (sensitivity: 0.875 and 1-specificity 0.220); (b) “Co-morbid behaviors” [AUC 0.720 (95% CI: 0.654–0.785), *p* < 0.001] optimal cut-off point 3.00 (sensitivity: 0.705 and 1-specificity 0.283); (c) “Co-morbid physical issues” [AUC 0.696 (95% CI: 0.629–0.763), *p* < 0.001] optimal cut-off point 3.00 (sensitivity 0.554 and 1-specificity 0.260) (Figure 3).

### 3.2. Reliability and Validity Measures for the APSI

The estimated internal consistency of the APSI was excellent (Cronbach alpha = 0.914). The split-half reliability technique showed that the APSI was internally consistent (split-half reliability coefficient = 0.847). The item scale correlations of the APSI 13 items ranged from 0.900 to 0.917. The internal consistencies of the APSI three factors were also excellent: (a) “Core autism symptoms” (Cronbach alpha = 0.910, item scale correlations 0.862–0.943); (b) “Co-morbid behaviors” (Cronbach alpha = 0.838, item scale correlations 0.763–0.808); and (c) Co-morbid physical issues” (Cronbach alpha = 0.729, item scale correlations 0.606–0.731). Finally, the test–retest stability was estimated between the first and the second administration of the APSI questionnaire. Precisely, a high degree of reliability was measured for the APSI total score with ICC equal to 0.922 [95%, CI: 0.900–0.940]; for the “Core Autism Symptoms” score with ICC equal to 0.948 [95%, CI: 0.933–0.960]; “Co-Morbid Behaviors” score with ICC equal to 0.855 [95%, CI: 0.813–0.888]; “Co-morbid Physical Issues” score with ICC equal to 0.838 [95%, CI: 0.792–0.875].

### 3.3. Principal Component Analysis (PCA) and Confirmatory Factor Analysis (CFA)

#### PCA Measures

Before running the principal component analysis, we investigated the correlation among the 13 items of the APSI questionnaire, and in our case, there was no need to exclude any item.

Factorability is better assessed using the Kaiser–Meyer–Olkin (KMO) method, which is also used to assess sampling adequacy. Kaiser’s recommendations propose using KMO 60 as a limit for assessing the factorability of the sample data. Based on this test, we could conduct a factor analysis because the overall KMO for our data was 0.87. Following that, we conducted Bartlett’s Test of Sphericity, and our estimated *p*-value of 0.001 showed that our data would benefit from a factor analysis. Additionally, our data matrix’s determinant was higher than 0, which suggests that our factor analysis would not likely encounter any numerical issues.

The factor loadings for each of the 13 items on the three factors, along with h2, which is the proportion of each variable’s variance that can be explained by the principal components (e.g., the underlying latent continua), are presented in Table 4. By inspecting the items included in each factor, the three factors expressed the following variables: factor 1, factor 2, and factor 3.

**Figure 4:**
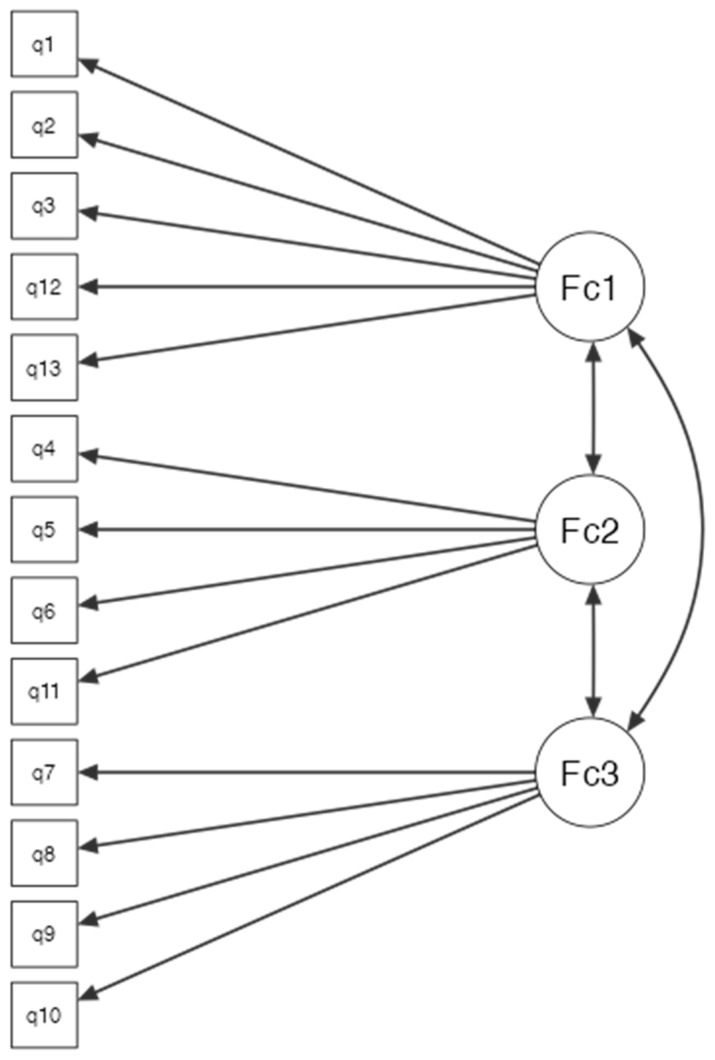
Visualization of the path diagram of the model (showing the standardized coefficients).

In the CFA, we used the results of the PCA. Namely, we investigated how well a CFA model fit the data in the three factors that the PCA suggested. Specifically, based on the PCA results for each factor, we only kept the items that had absolute (loading) > 0.4, so no question was excluded from the model. The analysis showed that our CFA model fit without any numerical issues. Figure 4 presents the path diagram of the estimated CFA model.

The summary statistics for this model were as follows: CFI = 0.886, TLI = 0.856, and RMSEA *p*-value = 0.128. These measures denoted an adequate fit of our data to the CFA model. The Cronbach’s alpha results were computed on the three new factors. The F1 and F2 had great internal consistency (alpha = 0.937) and (alpha = 0.828), respectively, while F3 had good internal consistency with alpha = 0.678. Finally, the pairwise Spearman rho correlation coefficient between the three factors was calculated. The analysis returned, reporting a statistically significant positive correlation between F1 and F2 (rs = 0.689, *p* < 0.001), F2 and F3 (rs = 0.547, *p* < 0.001), and F1 and F3 (rs = 0.547, *p* < 0.001).

## 4. Discussion

The present study aimed to validate the Greek version of the APSI among parents of children with ASD. A ROC analysis was used in the study to assess the discriminatory value of the scale. The results showed that the Greek version of the APSI was accurate and valid, in line with findings from previous validation studies, and Greek parents showed similar levels of parenting stress compared to parents in other countries [14,29,30].

Regarding the psychometric properties of the APSI, the ROC analysis revealed that the Greek version of the APSI showed discriminant validity for measuring parents with a child diagnosed with ASD. The calculated cut-off points between the ASD and TD groups were estimated at 12.00 (AUC: 0.839) out of a maximum score of 52.00 points. To our knowledge, only our validation study of the APSI calculated cut-off points derived from the scores’ percentile distribution [14,29,30]. Moreover, it should be noted that the Greek version of the APSI exhibited similar psychometric properties to the initial version and validation studies in the Chinese and Spanish languages [14,29,30]. The internal consistency was excellent (Cronbach’s α = 0.914). Also, the data analysis reported an acceptable range of Cronbach’s alpha values of its domains “Core autism symptoms” (a = 0.910), “Co-morbid behaviors” (a = 0.838), and Co-morbid physical issues” (a = 0.729). The test–retest stability was estimated between the first and the second administration of the APSI questionnaire. A high degree of reliability was measured for the APSI total score with the ICC equal to 0.922 [28].

A principal component analysis of the 13 items, using varimax rotations, was conducted, with the three factors explaining approximately 45.8% of the overall variance. All items had primary loadings over 0.61, except item 10. These results are consistent with the findings of another study [29], suggesting the universality of the questionnaire regardless of the culture in which it is implemented. Therefore, regarding internal consistency, the Greek version of the APSI was shown to be a valid scale and can be used as a reliable instrument for screening or quick assessment of parenting distress. However, regarding the principal component analysis (PCA) and confirmatory factor analysis (CFA), the results indicated that the failure to replicate the factors of the initial validation of the instrument should be kept in mind, and the instrument should be used in clinical practice with caution. This failure to replicate the factors was also indicated in the Chinese version of the APSI [29].

The study group consisted of parents of children with autism, and more specifically, 12 fathers and 101 mothers of children diagnosed with autism, covering a research gap in the literature about the value of an exclusive instrument to measure autism parenting stress [27]. The limited participation of fathers in the study was expected, as mothers usually are the primary parents, have the most active participation in the child’s activities, and bear the most significant burden [33,34,35,36,37]. According to our findings, it is worth mentioning that the initial validation study of the tool had a similar overall score to our findings, both in the ASD group and TD group, which shows the dynamics and reliability of the tool [14]. Another limitation of the study that should be noticed was the lack of divergent tests in the Greek language to compare with the APSI.

In addition, our study’s findings showed that the core symptoms domain recorded higher scores than the other two domains of the APSI. It is well known that core autistic symptoms include signal delays in and challenges with reaching self-regulatory milestones (tantrums, aggression, self-injurious conduct, and trouble transitioning; delays in appetite/digestion, sleep, and potty training) [14,38]. The symptoms mentioned above can cause a tremendous psychological burden in the daily lives of parents of children with autism [11,39]. Specifically, the core symptoms of children’s ASD were correlated with higher parenting stress and parental depression [11,39,40,41]. Moreover, our findings indicated a statistically significant difference between the ASD and TD groups regarding behavioral issues. It is well documented [41] that children’s behavioral issues, such as hyperactivity, aggression, self-harm, destruction (throwing or breaking objects), and disturbed mood, are strong predictors of parenting stress. Managing their children’s behavioral issues is a significant challenge for parents of children with ASD. Identifying these correlations and the additional factors that mediate them could serve as guidance for intervention programs.

## 5. Conclusions

In conclusion, the present study examined the Greek version of the Autism Parenting Stress Index. The Greek version of the APSI is a reliable and valid psychometric tool to measure the parenting stress that emerges from autism among parents with children with ASD. This instrument demonstrated excellent internal consistency, reliability, and validity. The findings are in accordance with the results from previous similar studies on other versions of the APSI across languages and cultures. Finally, the APSI can be practical for clinicians and researchers in Greece, providing a comprehensive evaluation of the autism parenting stress in daily practice.

The findings of this study must be seen in the light of some limitations. The primary limitation to the generalization of these results is the insufficient participation of male parents, as most of the sample comprised female parents (mothers). Future studies should replicate these findings in larger, gender-representative samples. Despite the above limitation, this study has significant value since there is a research gap in estimating the parenting stress of parents of children with ASD in Greece with a specific autism-related instrument.

Moreover, this study has significant implications for planning interventions for parents of children with an ASD diagnosis. A key goal of intervention programs for parents should be to enhance resilience and skills to manage challenging situations, as parents face continuous and various challenges. Research evidence demonstrates certain protective factors against parenting stress [42]. These factors include positive and problem-focused coping strategies, the family’s sense of control and self-efficacy, and perceived formal and informal social support. These factors explain why some families, despite the chronic stressors, have managed to adapt successfully and function with balanced dynamics. Thus, intervention programs should include counseling, education about autism, strategies for managing challenging behaviors, and methods to improve communication and social interaction, contributing to parents’ mental health and families’ quality-of-life improvements [43,44,45].

## Figures and Tables

**Table 1:** Demographics of the sample.

	Parents’ Group of ASD Children (*N* = 113)	Parents’ Group of TD Children (*N* = 127)	*p*
	*M* (*SD*) (Range)	*M* (*SD*) (Range)	
**Years of Age**	39.24 (6.70) (25–58)	41.08 (6.22) (27–56)	0.229 †
**Gender, *N***			
**Male**	12	24	
**Female**	101	103	0.102 ‡
**Level of** **Education, *N***			
**Junior High School**	6	3	
**High School**	56	73	
**University**	51	51	0.296 ‡
**Children’s** **Years of Age**	7.70 (4.40) (2–27)	7.77 (5.15) (2–22)	0.123 †

Abbreviations: TD, typical development; ASD, autism spectrum disorder; †, independent sample *t*-test; ‡, Pearson’s χ^2^ test.

**Table 2:** Between-groups comparisons of APSI total score and its three factors.

	Parents’ Group of ASD Children (*N* = 113)	Parents’ Group of TD Children (*N* = 127)		
	*Μ* (*SD*)	*Μ* (*SD*)	*t* (238)	*p*
**Core autism symptoms**	10.92 (4.28)	3.18 (4.37)	−13.823	<0.001
**Co-morbid behaviors**	4.82 (3.62)	2.44 (3.33)	−5.281	<0.0001
**Co-morbid physical issues**	3.67 (3.23)	1.94 (2.84)	−4.409	<0.001
**APSI total score**	19.39 (8.33)	7.59 (9.43)	−9.849	<0.001

Abbreviations: TD, typical developing; ASD, autism spectrum disorder; SD, standard deviation; APSI, Autism Parenting Stress Index.

**Table 3:** Group effects on the APSI total scores and its three factors.

	Parents’ Group of TD Children (*N* = 127)	Parents’ Group of ASD LEVEL 1 Children (*N* = 45)	Parents’ Group of ASD Level 2 Children (*N* = 38)	Parents’ Group of ASD Level 3 Children (*N* = 30)		
	*Μ* (*SD*)	*Μ* (*SD*)	*Μ* (*SD*)	*Μ* (*SD*)	*F* (3, 235)	*p*
**Core autism symptoms**	3.18 (4.37)	10.80 (4.42)	11.98 (4.24)	9.80 (3.92)	65.693	<0.001
**Co-morbid behaviors**	2.44 (3.33)	4.95 (3.89)	5.15 (3.41)	4.20 (3.51)	9.733	<0.001
**Co-morbid physical issues**	1.95 (2.84)	3.61 (3.51)	3.36 (2.89)	4.16 (3.28)	6.854	<0.001
**APSI total score**	7.59 (9.43)	19.27 (8.76)	20.47 (8.40)	18.16 (7.66)	32.552	<0.001

Abbreviations: TD, typical developing; ASD, autism spectrum disorder; SD, standard deviation; APSI, Autism Parenting Stress Index.

**Table 4:** Principal component analysis of the APSI questionnaire for the total sample.

Factor Items	Mean	Rotated Factor Loading			
		Factor 1	Factor 2	Factor 3	h^2^
**Factor 1**					
Question 1	1.40	**0.96**	−0.10	0.15	0.83
Question 2	1.37	**0.94**	0.23	−0.05	0.84
Question 3	1.14	**0.80**	0.13	−0.29	0.67
Question 12	1.60	**0.98**	0.36	0.32	0.88
Question 13	1.70	**0.92**	−0.52	0.07	0.82
**Factor 2**					
Question 4	0.93	0.15	**0.73**	0.05	0.61
Question 5	0.63	0.23	**0.92**	−0.09	0.79
Question 6	0.88	−0.38	**0.61**	−0.16	0.73
Question 11	0.74	0.15	**0.71**	−0.13	0.55
**Factor 3**					
Question 7	0.62	−0.02	−0.34	**0.66**	0.68
Question 8	1.01	0.09	0.22	**0.92**	0.70
Question 9	0.45	−0.11	−0.24	**0.70**	0.67
Question 10	0.71	0.23	0.06	**0.44**	0.35

Factor loadings above 0.40 are marked in bold.

## Data Availability

The data that support the findings of this study are available on request from the corresponding author. The data are not publicly available due to restrictions as they contain information that could compromise the privacy of research participants.

## Abbreviations

ASD	Autism Spectrum Disorders
TD	Typical Development
APSI	Autism Parenting Stress Index
QRS	Questionnaire on Resources and Stress
P-CDI	Parent–Child Dysfunctional Interaction
PD	Parental Distress
DC	Difficult Child
PSI-SF	Parenting Stress Index
SAC	Scientific Advisory Committee
M	Mean
SD	Standard Deviation
ROC	Receiver Operating Characteristics Curve Analysis
